# Refugees and access to employment in Brazil: implications for health and sociability

**DOI:** 10.1590/1980-220X-REEUSP-2023-0029en

**Published:** 2023-09-15

**Authors:** Jonas Sâmi Albuquerque de Oliveira, Marcelo Maurício da Silva, Mariana Mendes, Denise Elvira Pires de Pires

**Affiliations:** 1 Universidade Federal do Rio Grande do Norte, Departamento de Enfermagem, Natal, RN, Brazil.; 2 Centro Universitário do Rio Grande do Norte, Departamento de Direito, Natal, RN, Brazil.; 3 Universidade Federal de Santa Catarina, Programa de Pós-graduação em Enfermagem, Florianópolis, SC, Brazil.

**Keywords:** Refugees, Job Market, Right to Work, Right to Health, Refugiados, Mercado de Trabajo, Derecho al Trabajo, Derecho a la Salud, Refugiados, Mercado de trabalho, Direito ao trabalho, Direito à saúde

## Abstract

This is a reflective study with the objective of analyzing the possibilities of access to employment by refugees in the Brazilian territory, in view of the socio-legal conditions in Brazil, understanding the relationship between work and health. Refugees experience the penalty of restricted access to employment, which has a significant impact on their lives. By remaining disconnected from work, they are pushed to the margins of society. In addition to this, they face difficulties in accessing adequate housing, healthcare services, education, healthy food, among others, all of which deeply affect their way of life in a foreign country with a different culture. The work becomes relevant in the discussion about access to health, goods and services necessary to live with dignity, and its implications in the work-health-disease relationship. Collaborative actions at a global level are necessary to ensure that employment opportunities are available for refugees. In this regard, this reflection articulates two basic ideas: the recognition of the importance of work in social life and living conditions, and the understanding that the determination of the health-disease process goes beyond the biological and individual choices, having historicity and a social and cultural character.

## RESUMO

Trata-se de estudo reflexivo com o objetivo de analisar as possibilidades de acesso ao emprego por refugiados no território brasileiro, frente às condições sociojurídicas do Brasil, compreendendo as relações entre trabalho e saúde. Os refugiados vivenciam a penalização de acesso ao emprego com maior impacto em sua vida, pois ao manterem-se afastados do trabalho, são colocados à margem da sociedade. Somam-se a isto as dificuldades de acesso a moradias adequadas, aos serviços de saúde, educação, alimentação saudável, dentre outros, com implicações profundas no modo de viver em um país distinto do seu e de sua cultura. O trabalho adquire relevância na discussão sobre o acesso à saúde, bens e serviços necessários para viver com dignidade e suas repercussões na relação trabalho-saúde-doença, sendo necessárias ações colaborativas em nível global para garantir que o emprego esteja disponível para refugiados. Neste sentido, a presente reflexão articula duas ideias básicas: reconhecimento da importância do trabalho na vida social e nas condições de vida; e que a determinação do processo saúde-doença ultrapassa o biológico e as escolhas individuais, tendo uma historicidade e caráter social e cultural.

## RESUMEN

Se trata de un estudio reflexivo con el objetivo de analizar las posibilidades de acceso al empleo por refugiados en el territorio brasileño, frente a las condiciones socio-jurídicas en Brasil, comprendiendo la relación entre trabajo y salud. Los refugiados experimentan la penalización del acceso al empleo como el mayor impacto en sus vidas, ya que al mantenerse alejados del trabajo, se les coloca en los márgenes de la sociedad. A esto se suman las dificultades para acceder a una vivienda adecuada, servicios de salud, educación, alimentación saludable, entre otros, con profundas implicaciones en la forma de vida en un país y cultura diferentes. El trabajo adquiere relevancia en la discusión sobre el acceso a la salud, los bienes y servicios necesarios para vivir con dignidad y sus repercusiones en la relación trabajo-salud-enfermedad, requiriendo acciones colaborativas a nivel global para asegurar que haya empleo disponible para los refugiados. En ese sentido, esta reflexión articula dos ideas básicas: el reconocimiento de la importancia del trabajo en la vida social y en las condiciones de vida, y la comprensión de que la determinación del proceso salud-enfermedad va más allá de las elecciones biológicas e individuales, teniendo una historicidad y carácter social y cultural

## INTRODUCTION

The refuge situation in the world has been providing global debates about the integration of refugees into society in receiving countries, with extensive discussion on social policies that sometimes prove to be ineffective with regard to access to employment and personal and family subsistence production. Countries, together with multilateral organizations, have been mobilizing to address the refugee crisis, considered the most intense humanitarian crisis of the 21st century^(1)^.

At the international level, according to the report *Global Trends: forced displacement in 2022* ^(2)^, by the end of 2022, around 108.4 million people were forced to displace themselves. Within this huge contingent, there are about 35.3 million refugees, with low- and middle-income countries hosting 76% of this population. In the Brazilian reality, data collected by the National Committee for Refugees (Conare) and the United Nations High Commissioner for Refugees (UNHCR) indicate that, at the beginning of 2023, there were more than 65,000 people recognized as refugees in the country, of different nationalities, with a predominance of people of Venezuelan, Syrian, and Congolese origin^(3)^. The scenario of refuge in Brazil raises concerns about how these people settle and under what conditions they live in the country.

The ways in which people live and work, participation in the social structure, the guarantee of the right to health and access to available resources have a strong relationship with public policies, social justice, and the conception of the world and society^(4)^. The World Health Organization (WHO) and scholars in the field of health recognize the importance of building social policies that can ensure access to socioeconomic, environmental, and cultural conditions, life and work for equity in health^(5)^, which includes people as refugees. The United Nations (UN), when defining the 17 Sustainable Development Goals (SDGs), also relates life, decent work, health and well-being^(6)^, and society must recognize the importance of full enjoyment of social rights by refugees. Refugees and social rights, including the right to decent work and health, are highly relevant issues for the human and Brazilian societies, especially for health professionals, managers, and public policy makers.

In this context, Brazil has shown, over the last few decades, greater interest in resolving the social inequities experienced by refugees. Law No. 9.474/1997^(7)^, known as the Refuge Law, is considered one of the most advanced standards on the subject in the country and is inspired by the 1951 United Nations Convention^(8)^. The purpose of the Convention was to ensure the broadest possible exercise of human rights and fundamental freedoms, recommending compliance with such measures by States. It is a document that consolidated several international legal instruments and implemented a text that lists the rights of refugees at an international level^(8)^.

In the Brazilian law^(7)^, the definition of a refugee includes the reason for determining refugee status, the origin from a region with serious and generalized violation of human rights. This definition was strongly inspired by the Cartagena Declaration of 1984, which dealt with the discussion on asylum and international protection of refugees in Latin America^(9)^. Brazil also adopts the recommendations when welcoming refugees in the country, including them in the list of rights and duties, with many similarities to the native peoples in the nation.

People in a situation of refuge become vulnerable populations, with difficulties in accessing health services, social programs of embracement, and work environments free from harassment, suffering, and discrimination^(10,11,12,13)^, with the support and intervention of the State being fundamental. Likewise, collaborative actions are needed at the global level to ensure that employment is available for refugees, coinciding with what is advocated about the right to health and the relationship between life, social rights, and health of individuals and populations. The characteristics of the labor market in the globalized world, the specific realities of the economic conformation experienced by the countries and the influencing scenarios of changes in the supply and demand of jobs have interfaces with the way refugees work and live.

In this respect, the present reflection articulates two basic ideas: the recognition of the importance of work in social life and living conditions, and the understanding that the determination of the health-disease process goes beyond the biological and individual choices, having historicity and a social and cultural character. This is relevant in analyzing the possibilities of employment access for refugees welcomed in Brazilian territory, in what regards the social-legal conditions in Brazil.

The study was built upon the premises of public international law, as it seeks to identify the normative option of the Brazilian State regarding the integration of refugees in the labor market, with a view to the humane embracement of these groups, as well as ensuring their essential right to full citizenship and universal access to health. The legal problem of this study focuses on International Refugee Law (IRD), more specifically on the analysis of the possibilities of ratification (internalization or nationalization) of the assumptions and recommendations of the UN regarding access to employment for refugees in the Brazilian territory. Furthermore, the theoretical foundation for the analysis of the work category was dialectical historical materialism^(14)^, justified by the need to understand the complexity of the object of study related to the situation of the labor market for the refugee population in Brazil. To understand the relationship between work and health, a dialogue was held between the theoretical model proposed by Dahlgren and Whitehead^(5)^, which deals with the Social Determinants of Health (SDH), adopted by the WHO, and the theoretical model of the Social Determination of the Health-Disease Process by authors of the Latin American Collective Health^(4)^. The SDH model understands the determinants arranged in different layers, with the closest layer occupied by individual determinants and a distal layer with macro-determinants, where living and working conditions, work environment, and unemployment are located. Theorization of the Social Determination of the Health-Disease Process considers the historicity of these elements and the nature of the process in determining social issues.

Thus, this reflection aims to analyze the possibilities of access to employment by refugees in the Brazilian territory, in view of the socio-legal conditions in Brazil, understanding the relationship between work and health.

### Work in the Capitalist Society: Interfaces With the Workforce Exploitation

Work plays a fundamental role in life in society. As a conscious and purposeful human activity, it constitutes a force capable of transforming the world we live in and know. Working, the human being creates him/herself, and in this act establishes a rupture with their natural state, giving rise to the social being. Human work can be understood as effort and result^(14)^.

In the capitalist society logic, the employer is the owner of the means of production and controls the production processes, so that, tendentially, the worker is left with the only alternative for sustaining their life - the sale of their labor power^(14)^. Even with the profound changes in society, since the emergence of the capitalist mode of production, the logic of relations between capital and work influences the ways of producing in the various sectors of the economy. On the other hand, when selling their workforce, in addition to seeking conditions for their survival, the worker also seeks to give meaning to their existence, to feel useful and part of collectives.

The Taylorist-Fordist model of development, which emerged after the Second World War and was particularly prominent in the major capitalist economies, entered into crisis in the late 1960s. From the 1970s and 1980s onwards, there were profound transformations in the ways of organizing production and in the structure of companies, made possible by strong technological development, as well as changes in the understanding of the role of the State in the economy, leading to substantial deregulation of relations between capital and labor. This phenomenon was called productive restructuring^(15)^, with remarkable characteristics in each historical context, mediated by the influences and negotiation power of political, social, and economic agents.

The flexibilization of labor relations, in practice, means an erosion of relatively contracted and regulated work, heir to the Taylorist and Fordist era^(16)^. The work model that stood out in the 20th century, particularly during the so-called “glorious 25 years,” was strongly influenced by the workers struggle for social rights. However, from the last decades of the century onwards, this model has been replaced by various forms: entrepreneurship, cooperativism, volunteer work, atypical employment forms that oscillate between overexploitation and the self-exploitation of labor^(16,17)^.

The 21st century is characterized by the intense process of technological innovations, the globalization of production and communication, but also by setbacks in relation to labor rights, environmental and climate crises, as well as wars, violence, and social exclusion. There is the emergence and resurgence of endemics and epidemics, including a pandemic that took the lives of millions of people^(1)^. The impact on society is enormous, causing and being affected by migratory flows of people, including those who are refugees.

When considering the ramifications of the situation of the quality of work, with regard to labor protection, there is a direct relationship between the right to work for refugees and possible access. In the contemporary world, specifically, there is a situation of labor rights that occurs autonomously, with no direct relationship with struggles for better wages, as well as in employment situations in the spheres of private or public services^(17)^.

This sad reality is related to the process of globalization of capital, with consequences in the destruction of jobs and the creation of new ones. This is a phenomenon with direct interference from the flexibility of capital, which can invest or disinvest at any time in any market, regardless of nationality, with the clear intention of accumulating and offering advantages^(18)^. The conditions to which workers are currently exposed, under the hegemony of capitalist logic, move towards a structural precariousness of the workforce on a global scale. Added to this is the situation of the explosion of unemployment that affects huge contingents of workers, such as men or women, stable or precarious, formal or informal, natives, immigrants, and refugees.

Unemployment can limit the integration of refugees. On the other hand, obtaining a job is considered a key factor for a successful integration into society, coinciding with what is advocated by the SDH^(13,19)^. The insertion of refugees at work has interfaces with the challenges faced in establishing themselves in the labor market of the host countries. This includes issues as traumas related to experiences lived in scenarios of conflicts, violence, and difficulties, physical and mental health problems, difficulties with language and to establish new relationships, as well as sociocultural aspects^(19,20)^.

In this situation, the refugee population becomes doubly vulnerable, either due to their lack of employment opportunities and/or because they may be subjected to exploitation when entering the labor market. It is, therefore, essential to reflect on the availability of jobs that allow access to the goods and services required to live with dignity. Briefly, refugees experience the penalization of access to employment with the greatest impact on their lives, as by staying away from work, they are placed on the margins of society. Moreover, there are the difficulties in accessing adequate housing, health services, education, healthy eating, among others, with great impact on the immigrants’ way of life in a different country and culture.

### Brief Contextualization of the Protection of Refugees in the Brazilian Territory

The first decades of the 21st century are marked by a new major cycle of transformations in the ways of producing, with an intensification of the technological innovation process, especially digital technologies. On the one hand, we have the frenzy of instant communication, industry 4.0 and 5.0, the internet of things, Artificial Intelligence (AI) and, while on the other hand, there is a deepening of inequalities and a significant number of people in situations of internal, foreign and stateless displacement in various parts of the world. In addition, consideration should be given to including people in a refugee situation who do not have a nationality, as well as those who are outside the country in which they had their usual residence. This condition is justified by the impossibility of returning to their country of origin, due to fear of insecure situations that countries present for this specific population, as well as the spontaneous desire not to return to that State.

Considering the Geneva Convention, the definition of refugee refers to any person who, fearing persecution for reasons of race, religion, nationality, social group or political opinion, is outside the country of his nationality and cannot or, due to this fear, does not want to avail him/herself of the protection of that country^(8)^. In the Brazilian standard, to the definition of a refugee is added to be a victim of serious and generalized violation of human rights^(7)^.

It can be seen that the causes of refuge are multifactorial and complex, and the support of entities and bodies capable of directing actions is required, with a view to guaranteeing their rights. At the international level, an important milestone was the creation of UNHCR, in 1950, which today is a permanent subsidiary agency of the General Assembly of the United Nations, based in Geneva. UNHCR is responsible for ensuring the application of international conventions that ensure the protection of refugees^(2)^.

After its creation, the International Convention Relating to the Status of Refugees was held in 1951, aiming at revising and codifying previous international agreements relating to the status of refugees and expanding international cooperation agreements and relations for the protection of populations in situations of refuge^(8)^. In Brazil, in 1960, the Refugee Convention was ratified, with the enactment of Decree No. 50.215. It should be noted that although Brazil adhered to the Convention in the 1960s, UNHCR only established its presence in Latin America 20 years later, and with important actions undertaken only in Central America, maintaining little activity in South America.

In the following years, around 1970, Brazil and almost all of South America experienced a sequence of regimes of exception, with dictatorships that forced thousands of citizens to leave abroad. During this period, UNHCR made an important international contribution in monitoring situations of human rights violations.

It was only later that one of the biggest steps towards this protection was envisioned, the edition of the Brazilian Refugee Protection Law no. 9.474 of 1997, which discussed the main rights of asylum seekers and refugees in the country^(7)^. Based on this norm, there were changes in the dimension of the study of International Refugee Law in Brazil. In 2022, the 25th anniversary of its enactment was celebrated, with a broad debate among the various social and political actors about the advances and challenges of the situation of refuge in the country and the guarantee of the refugees’ rights.

From this perspective, we recall that although there have been advances in refugee legislation in Brazil, during the crisis of asylum seekers in the country (Haitian people, in the period of 2012 and 2013), the situation did not develop with the expected reception, leaving the route of the ‘humanitarian visa’. Added to this is the recognition of the legal situation of refuge for thousands of Venezuelans in 2019. This fact prompted the country to analyze the challenges it will have to face in this century regarding the issue of welcoming refugees, considering Brazil’s growth in the world economic scenario, as well as the constant search for refuge by people from different continents^(3,8)^.

Based on the above, it is possible to see Brazil’s progress towards refugee rights; however, protection strategies are still incipient in view of the demands of this population. Under this analysis, in February 2023, Ordinance No. 70 was published, which defines the composition of a multisectoral Working Group to prepare the National Policy on Migration, Refuge and Statelessness. This National Policy will aim to articulate actions in five axes: migratory regularization; local integration; promotion and protection of rights; fight against xenophobia and racism; social participation; and international relations and interculturality^(21)^.

Attempts to expand the role of the Brazilian State in the face of the paradox of migration and refugee processes demonstrate the movement of society and organizations to ensure that the refugee population has their rights guaranteed. The existence of an updated policy allows the development of more assertive, efficient and humanized strategies, with the sole objective of guaranteeing that refugees in Brazil will have their voices heard and needs met.

### Access to Work by Refugees in Brazil: Challenges and Limits

Access to employment by refugees in Brazil occurs in the context of the metamorphoses in the current world of work, including requirements and availability of absorption in the market, associated with the scenario of guarantees of legal, international and national protection. Multiple partners and sectors of society have made efforts to ensure the inclusion of the refugee population in public and private services and programs in the country. We highlight access to employment and income generation, and the stimulation and involvement of the private sector, universities and development actors with actions aimed at the full social and economic integration of the population in refuge in Brazil.

The concerns with the provision of jobs to refugees is global and leads to even deeper and more complex tensions, such as the receiving countries’ responsibility in relation to the insertion of these people in society, in particular, at work. This occurs, in part, due to the economic impact produced by mass migratory flows on the local economy, due to the need to reallocate migrants and refugees in the labor market, to contribute to the economic development of the host country.

This situation is aggravated by the fact that refugees are unable to use their professional skills, even in situations of expansion of the labor market and availability of jobs. It should be noted that one of the major obstacles faced by this population seeking insertion in the receiving country is the difficulty in validating their higher education certificates, making it difficult to prove the qualifications obtained in training and experiences in the country of origin^(12,19)^.

This situation of entering labor activities after refuge is quite emblematic and dramatic, leading to a migratory process that degrades, disqualifies the refugee socially and economically^(10–13,19,22)^. In addition, there are other obstacles, such as the lack of domain of the Portuguese language, the lack of documents, racial prejudice, and lack of resources to look for a job, since there are expenses with displacement, food and document printing during the search for the job. Faced with these conditions of the refugee population, the Brazilian labor market has become increasingly challenging and restricted.

To face this situation, some initiatives developed by UNHCR and partners stand out, which are expressed in Brazil, aiming to integrate and provide opportunities for refugees to be inserted in formal work. One of them, developed by the Companies with Refugees Forum, aims to promote the exchange of experiences between companies that present training actions for the purpose of inclusive hiring^(23)^. Another is the Entrepreneurial Refugees (*Refugiados Empreendedores*) initiative, which offers visibility to businesses led by refugees, encouraging the purchase of their products^(23)^.

In addition, there is an initiative aimed at work and employment relations, the Protect Work Campaign (*Campanha Proteja o Trabalho*)^(24)^, meant for presenting important information about the measures adopted in labor and employment relations in Brazil. It is also possible to report slave labor on the website. The site content is presented in Portuguese, Spanish, English, French and Arabic, to broaden access to information.

Considering the significant increase in refugee women in Brazil, some initiatives are specific to guarantee their empowerment and employability. The Program *Moverse* promotes the access of Venezuelan refugee and migrant women to the Brazilian labor market, considering everything from training to raising awareness of the private sector^(23)^. Another initiative is Empowering Refugees (*Empoderando Refugiadas*), which seeks to promote the employability of women hosted in emergency shelters in Boa Vista, in Roraima, with a special focus on the inclusion of LGBTIQ+, disabled, and over 50 years old women^(23)^.

All these initiatives presented, among others, emphasize the importance of work in the lives of refugees and call attention to the development of strategies to face this problem. Brazil has sought to guarantee access to jobs in the various regions of the country. A successful example is the Interiorization Strategy (*Estratégia de Interiorização*), which takes refugees from Roraima to around 800 Brazilian municipalities in search of new opportunities, with special attention to combating gender inequalities and discrimination^(25)^.

UNHCR and UN Women, in partnership with the Universidade Federal de Goiás, carried out a survey in 2021 with interviews with more than 2,000 people involved in the Interiorization Strategy and the results show that single people without children end up being more likely to go to other states with a possible job vacancy. The survey also points out that there is greater difficulty in labor insertion among women, especially for those with many children and single-parent families. In general, it can be seen that female participation of refugees in the labor market is lower (72.2%) when compared to men (96.1%) and unemployment rates are higher among women (17.7%) than among men (6.4%). Rates among women are also higher in what regards level of labor informality (37.3%), being 1.2 times higher than that of men (29.4%)^(25)^. This is concerning due to the expression of gender inequality, aggravated by the implications for survival, education, and access to health resources for children cared for by mothers in this condition.

Similarly, a study carried out in Africa highlights how the structural conditions of refugees’ lives can restrict the right to work or seek education, lead to ruptures in social relations, and give rise to discrimination/inequity and gender violence. The consequence of being subjected to discrimination, inequality and violence is poor mental health and psychosocial well-being^(22)^, as well as problems with food, shelter and access to health.

Data from the aforementioned surveys reinforce the importance of countries being aligned in their perspectives related to refugees. The awareness to serve vulnerable populations has been signaled worldwide, such as the 169 goals that make up the 2030 Agenda, which are aimed at achieving the SDGs, and apply to the phenomenon of refuge^(6)^. In light of this, the search for compliance with existing legislation is constant, aiming to ensure the embracement of the refugee population, as well as the production of specific public policies with a view to the fundamental guarantees of life in the Brazilian society.

### Work and Implications on Refugees’ Health

Refugees’ access to employment in Brazil is part of the context of difficulties faced by people who arrive in the country in search for better living and health conditions. Work, when considered the way for human beings to guarantee their subsistence in the capitalist society^(14)^, has the potential to produce satisfaction and promote health, or become a disease-causing factor. On the other hand, work is also a privileged locus for building social identity^(26)^, impacting the way refugees experience life and feel they belong to the host country.

The way people live and work is related to their health status^(4,5)^. In this context, the work acquires relevance in the discussion about the access and right to health of refugees in Brazil. The work-health-disease relationship is both recognized in theoretical perspectives, such as the one formulated by Dahlgren and Whitehead^(5)^ and assumed by the WHO, and expressed in Brazilian documents and legal framework^(27)^ and in theories about the social determination of the health-disease process^(4,5)^.

The WHO acknowledges that health is more than the absence of disease and that it is determined by factors related to living and working conditions, availability of food, and access to essential environments and services, such as health, housing, water and sewage, employment and education. These factors are interconnected, indicating the importance of favorable living conditions for maintaining the health of populations^(4,5)^.

In the Brazilian context, the text of the 8th National Health Conference, in 1986, recognizes the relationship between health and social rights, including access to work and employment. The text approved at the conference expresses, textually, that health is not an abstract concept and that it is expressed in undifferentiated subjects.

In its broadest meaning, health results from the conditions of food, housing, education, income, environment, work, transportation, employment, leisure, freedom, access and ownership of land, and access to health services. It is therefore, above all, the result of forms of social organization of production, which can generate great inequalities in living standards. Health is not an abstract concept. It is defined in the historical context of a given society and at a given moment in its development, and must be conquered by the population in its daily struggles^(27:4)^.

In the Brazilian Constitution of 1988, health is understood as a universal right, “everyone’s right and duty of the State, guaranteed through social and economic policies aimed at reducing the risk of disease and other injuries and universal and equal access to actions and services for their promotion, protection and recovery”^(28:118)^. Understood as a right and determined by the conditions in which people live and work, health is directly related to State policies. Work and health are rights of refugees and must be guaranteed throughout the national territory.

Work is fundamental to providing and guaranteeing human subsistence; therefore, policies must be monitored by the different governmental agencies, to map the key points that influence the health-disease situation and act strongly on them. Most refugees are in vulnerable situations. These people end up exposed to conditions of profound social inequality, including difficulties in entering the formal job market, which is currently aggravated by an adverse scenario of precarious work relationships and unemployment.

The refuge phenomenon is complex and multifaceted and, in general, involves several cultural issues and violation of rights in the home countries. Within this frame of reference, it is believed that people who are forced to move, especially refugees, are already in a situation of vulnerability in their own country and return to live in unfavorable conditions in the country of destination. Therefore, it is essential that social and health policies guarantee efficient regulatory measures, as well as healthy and favorable environments for the life and work of refugees.

International organizations, together with countries and civil society, need to invest in strategies that guarantee this population’s access to employment, food, health and education, among others. It is necessary to combat any type of prejudice related to the refugee population, with an emphasis on issues of gender identity, race, and age^(29)^. In the labor market, it is essential to value individual skills, prior training, and interest in learning, ensuring legal recognition of the rights of refugees, especially with regard to their right to work^(30)^.

Ensuring decent, regulated, safe and healthy work has the potential to improve the living conditions of refugees. Combating the informal market and exploiting this population’s workforce has to be a constant agenda for interventions, seeking their full integration into the country’s work activities. Brazil has several areas of economic activity, being able to absorb workers with different qualifications, skills and abilities. Thus, it is essential that the measures to protect refugee workers are complied with and that their work in the formal labor market encouraged.

This study contributes to the field of nursing by shedding light on the interfaces between work and health for vulnerable populations, which include refugees. The reflection points to one of the central aspects of the principles of the Brazilian Public Health System – intersectoriality, which provides for the articulation of the health sector with other areas of society to act in the face of social and health conditions and determinants, with the potential to have a positive impact on life of people in refugee status. We emphasize that the development of strategies to integrate refugees into the Brazilian labor market must involve combating the exploitation of the workforce and informality, eradicating all types of violence, prejudice, and discrimination, valuing previous skills, training in/for work, and allowing favorable and healthy relationships at work and in other social environments. In summary, we reflect that access to employment is a viable alternative to promote access to social benefits, and this includes the possibility of eating and living adequately, with implications for the refugees’ health-disease condition.

The limitations of the study involve the fragmentation of data related to the situation of refugees, their conditions of access to work, and the consequences for their health and life. It is observed that, even with the advances in the Brazilian legislation regarding the acceptance of the recommendations of international organizations for the care of refugees, the findings that supported this study translate into specific initiatives of States and regions. They fail to translate a concern of the country in all its territorial dimension.

## Final Considerations

The debate on access to employment by refugees in the Brazilian territory, including the analysis of socio-legal conditions in Brazil and the relationship between work and health, is challenging, as it is related, in the first place, to the priorities for adopting legal compliance strategies of recommendations from international organizations.

Added to this are the forms of receiving and embracing people in this condition in the labor market of a country that is increasingly metamorphosed from conditions that impose the deregulation of the work of people with Brazilian nationality, including precarious work and underutilization of the refugee worker’s skills and competences.

SDHs are guiding points of political and social strategies, in which work acquires relevance due to its potential to improve the way refugees live in the country, with emphasis on maintaining their subsistence and access to goods and services necessary to live with dignity. The work provides relations of belonging of the refugee to the nation that embraced them and stimulates the development of skills and competences necessary to carry out the work activity.
